# T Cell Responses Induced by DNA Vaccines Based on the DENV2 E and NS1 Proteins in Mice: Importance in Protection and Immunodominant Epitope Identification

**DOI:** 10.3389/fimmu.2019.01522

**Published:** 2019-07-03

**Authors:** Paolla B. A. Pinto, Maysa L. Assis, Adriana L. Vallochi, Agatha R. Pacheco, Lauro M. Lima, Kátia R. L. Quaresma, Bernardo A. S. Pereira, Simone M. Costa, Ada M. B. Alves

**Affiliations:** ^1^Laboratory of Biotechnology and Physiology of Viral Infections, Instituto Oswaldo Cruz, Fundação Oswaldo Cruz (Fiocruz), Rio de Janeiro, Brazil; ^2^Laboratory of Immunopharmacology, Instituto Oswaldo Cruz, Fundação Oswaldo Cruz (Fiocruz), Rio de Janeiro, Brazil

**Keywords:** dengue, DNA vaccines, T cell response, NS1, envelope protein, mice

## Abstract

The importance of the cellular immune response against DENV has been increasingly highlighted in the past few years, in particular for vaccine development. We have previously constructed two plasmids, pE1D2, and pcTPANS1, encoding the envelope (E) ectodomain (domains I, II, and III) and the non-structural 1 (NS1) protein of dengue virus serotype 2 (DENV2), respectively. In the present work, we analyzed the induction of the cellular response in mice immunized with these DNA vaccines and identified the immunogenic peptides. Vaccinated BALB/c mice became protected against a lethal challenge of DENV2. Depletion of CD4^+^ cells in vaccinated animals almost completely abolished protection elicited by both vaccines. In contrast, a significant number of pE1D2- and pcTPANS1-immunized mice survived virus challenge after depletion of CD8^+^ cells, although some animals presented morbidity. To identify immunogenic peptides recognized by T cells, we stimulated splenocytes with overlapping peptide libraries covering the E and NS1 proteins and evaluated the production of IFN-γ by ELISPOT. We detected two and three immunodominant epitopes in the E and NS1 proteins, respectively, and four additional NS1-derived peptides after virus challenge. Characterization by intracellular cytokine staining (ICS) revealed that both CD4^+^ and CD8^+^ T cells were involved in IFN-γ and TNF-α production. The IFN-γ ICS confirmed reaction of almost all E-derived peptides before challenge and identified other epitopes after infection. All NS1-derived peptides were able to elicit IFN-γ production in CD4^+^ cells, while only a few peptides induced expression of this cytokine in CD8^+^ T lymphocytes. Interestingly, we observed an increase in the frequency of either CD4^+^ or CD8^+^ T cells producing TNF-α after immunization with the pE1D2 and challenge with DENV2, while lymphocytes from pcTPANS1-vaccinated animals maintained ordinary TNF-α production after virus infection. We also assessed the recognition of E and NS1 immunogenic peptides in C57BL/6 mice due to the difference in MHC haplotype expression. Two NS1-derived epitopes featured prominently in the IFN-γ response with cells from both animal strains. Overall, our results emphasize the importance of the T cell response involved in protection against dengue induced by E and NS1 based DNA vaccines.

## Introduction

Dengue is one of the most important mosquito-borne viral diseases, with an overall estimation of 390 million people infected worldwide per year (1). This disease can manifest as a broad range of symptoms varying from the self-limiting dengue fever to potentially lethal severe forms, the dengue hemorrhagic fever, and dengue shock syndrome (2, 3).

Currently, different vaccines against dengue virus (DENV) are undergoing clinical trials and one is commercially available (4–6). This tetravalent vaccine, developed by Sanofi-Pasteur, is based on the backbone of the yellow fever 17D vaccine with the replacement of membrane and envelope proteins of each dengue serotype (7). Results from phase III clinical trials revealed that although individuals, especially children, presented high levels of neutralizing antibodies toward DENV2 after vaccination, they were not protected against this virus (8–10). Furthermore, recent reports showed that children who were dengue naïve at vaccination time were more susceptible to develop severe dengue after virus exposure (11–13). Such results point out that the induction of neutralizing antibodies is not the only arm of the immune response involved in protection against DENV. In fact, the importance of the cellular immune response against DENV has been increasingly highlighted in the past few years, concerning both protection and/or disease enhancement (14–20).

Most vaccine strategies against dengue are based on the envelope (E) glycoprotein. The E protein is organized in 90 homodimers in the virus surface and is reassembled into trimers at the fusion state. Each monomer is composed of domains I, II, and III, a membrane-proximal stem and a transmembrane anchor (21). Since the E protein is the major component of the virion surface and interacts with receptors present on host cells mediating virus internalization, this protein is the primary target for induction of neutralizing antibodies (22–24). However, after virus infection a T cell response that may be involved in protection is also elicited toward this protein (25–30).

Another highly immunogenic dengue protein is the non-structural 1 (NS1) glycoprotein, which is also considered an antigen for vaccine development (31–34). The NS1 is found in infected mammalian cells associated with plasma membrane as well as secreted into the circulation as soluble multimers (35–37). The secreted form seems to be implicated in immune evasion strategies (38). Moreover, convalescent dengue patients present high levels of antibodies against NS1. Recent studies also showed that this protein can induce T cell responses either in experimental animals or in humans (27, 29, 30, 39).

Based on this evidence, in the present work, we analyzed the induction of the cellular immune response in mice immunized with two previously constructed DNA vaccines (pE1D2 and pcTPANS1) against DENV2. The plasmid pE1D2 encodes the ectodomain of the E protein (domains I, II and III) (40, 41), and the plasmid pcTPANS1 contains the *ns1* gene (33, 42). BALB/c mice immunized with these DNA vaccines became protected against a lethal challenge of DENV2 and we evaluated the role of T cells in protection. Depletion of CD4^+^ T cells in vaccinated animals completely abolished protection elicited by both vaccines, while a significant number of pE1D2- and pcTPANS1-immunized mice survived virus challenge after depletion of CD8^+^ T cells. However, near half of the vaccinated animals depleted from CD8^+^ T cells presented clinical signs of infection.

We then identified the immunogenic peptides recognized by T cells from vaccinated animals, before and after virus challenge. We evaluated IFN-γ production by stimulating splenocytes with overlapping peptide libraries covering the E and NS1 proteins in enzyme-linked immunospot (ELISPOT) assays. Intracellular cytokine staining (ICS) assays revealed the involvement of both CD4^+^ and CD8^+^ T cells in IFN-γ production by splenocytes reacting mainly to the selected E-derived peptides, before and after virus challenge. In contrast, almost all the selected NS1-derived peptides were recognized by CD4^+^ T lymphocytes, while CD8^+^ cells reacted only to half of these peptides. TNF-α production was also evaluated by ICS assays, revealing expression of this cytokine by CD4^+^ and CD8^+^ T cells upon stimulation with almost all E-derived peptides, especially after virus challenge. On the other hand, only two NS1-derived peptides were able to significantly induce TNF-α production. To assess the recognition of immunogenic E- and NS1-derived peptides in a different MHC haplotype, we investigated IFN-γ production in C57BL/6 mouse cells by ELISPOT. Distinct epitopes were recognized by C57BL/6 splenocytes using the same peptide libraries, although two NS1-derived epitopes were positive in the assays performed with cells from both BALB/c and C57BL/6 animals. Overall, our results emphasize the importance of the T cell response involved in dengue protection and may contribute for the development of more effective vaccines against dengue, in particular for DNA vaccines based on the E and NS1 proteins.

## Materials and Methods

### Virus and DNA Vaccines

The dengue 2 virus (DENV2), strain New Guinea C (NGC, GenBank M29095), was used for cloning the NS1 and E sequences as well as for mice challenge assays. The DNA vaccines pcTPANS1, pE1D2, and pcTPA were previously described (33, 40). Briefly, the pE1D2 plasmid encodes the ectodomain (domains I, II, and III) of the E protein while pcTPANS1 encodes the full-length *ns1* gene. In both constructions, genes were fused to the sequence encoding the human tissue plasminogen activator (t-PA) signal peptide, under the control of the cytomegalovirus (CMV) promoter region. The negative control, pcTPA, was derived from the commercial vector pcDNA3 (Invitrogen) and contains only the t-PA signal peptide sequence.

For DNA vaccine preparations, *Escherichia coli* DH5-α strain was transformed with the different plasmids, which were then extracted by alkaline lysis and purified by Qiagen Endofree Plasmid Giga Kit (Qiagen), according to manufacturer's instructions. Plasmids were suspended in sterile water and stored at −20°C until use. The DNAs were quantified by measuring absorbance at 260 nm in spectrophotometer (Bio Photometer, Eppendorf). Concentration and integrity of all plasmids were confirmed by 1% agarose gel electrophoresis, stained with Nancy-520 and visualized in ultraviolet transilluminator.

### Animal Immunization

Four-week-old BALB/c or C57BL/6 mice, specific pathogen free (SPF), were purchased from the Multidisciplinary Center for Biological Investigations (CEMIB, UNICAMP-SP, Brazil). Animals were inoculated by the intramuscular route (i.m.) with 50 μg of DNA vaccines diluted in 50 μL of phosphate buffer saline (PBS) in each tibialis posterior muscles (100 μg/mice), using 30-gauge needles. Each animal group received two doses of pE1D2 or pcTPANS1, administered 2 weeks apart, and mice were euthanized or challenged 4 weeks after the first dose. Negative control groups included naïve or pcTPA-inoculated mice. For euthanasia, animals were overexposed with a mixture of ketamine-xylazine (43) and bled by cardiac puncture.

### Virus Challenge

BALB/c mice were anesthetized with a mixture of ketamine-xylazine and inoculated by the intracerebral (i.c.) route with 30 μL of a neuroadapted NGC DENV2 diluted in E199 medium, corresponding to 40 LD_50_. Animals were euthanized either 7 or 21 days after infection (dpi) as described on figures legends. To ascertain vaccine protection, mice were followed up to 21 dpi, and morbidity was recorded. Clinical signs of infection were noted according to an arbitrary scale ranging from 0 to 4: 0 = no clinical signs; 1 = paralysis in one leg or alteration of the spinal column; 2 = severe paralysis in one leg and alterations of the spinal column or severe paralysis on both hind legs; 3 = severe paralysis in the hind legs and alteration of the spinal column; 4 = death. Moribund animals were submitted to euthanasia.

### *In vivo* Depletion of CD4^+^ and CD8^+^ Cells

BALB/c mice (*n* = 8–10) immunized with pE1D2 or pcTPANS1 were depleted from CD4^+^ or CD8^+^ cells upon inoculation of in-house produced ascitic fluids containing anti-CD4 (clone GK 1.5) or anti-CD8 antibodies (clone 56-3.7) (39). Animals were inoculated by the intraperitoneal route (i.p.) with 20 μL of ascitic fluids on days 4 and 2 before DENV2 challenge. T cell depletion was monitored by flow cytometry in blood cells stained with anti-CD3 FITC (clone 145-2C11), anti-CD8 PerCP (clone 33-6.7) and anti-CD4 PE (clone RM4-5) antibodies (BD Biosciences), and evaluated on FlowJo software.

### E and NS1 Peptide Libraries

The T cell epitope mapping and characterization were performed using overlapping peptide libraries spanning the E and NS1 protein sequences of DENV2 NGC strain, consisting of 15-mer peptides overlapping each other by 11 amino acid residues. Lyophilized synthetic peptides (Mimotopes Pty Ltd) were suspended in ultrapure water at a final concentration of 2 μg/μL, with an average purity of ~80%. After the suspension, peptides were stored at −20°C until use. Initial studies were carried out with peptides grouped in pools of 8–10 peptides according to each protein (E protein: 98 peptides, 10 pools; NS1 protein: 86 peptides, 9 pools).

### Cell Isolation

Spleens and peripherical blood were harvested from previously pE1D2-, pcTPANS1- or pcTPA-inoculated animals, challenged or not with DENV2, as well as from naïve mice. For ELISPOT and ICS assays, splenocytes were isolated, and erythrocytes were lysed with BD Pharm Lyse™ (BD Biosciences), according to the manufacturer's instructions. After red cell lysis, splenocytes were washed with PBS and suspended in RPMI-1640 medium (Sigma), in 10% fetal calf serum (FCS), 100 IU/mL penicillin, and 100 ug/mL streptomycin. Blood samples from T cell-depleted mice, collected at the same day of virus challenge in the other animal groups, were treated with FACS Lysing Solution (BD Biosciences) for erythrocytes lysis and cell fixation, prior to staining and flow cytometry analysis.

### Interferon Gamma ELISPOT Assays

The assays were performed using the IFN-γ ELISPOT mouse set (BD Biosciences) upon splenocyte stimulation with E and NS1 peptide libraries, according to the manufacturer's instruction. The assays were initially performed using pooled peptides. Afterwards, positive pools were selected for screening of individual peptides. Briefly, 96-well ELISPOT plates were coated overnight at 4°C with anti-IFN-γ capture monoclonal antibody (5 μg/mL). On the next day, plates were blocked and 5 x 10^5^ splenocytes (*n* = 3, pooled cells) from naïve, pE1D2- or pcTPANS1-vaccinated or pcTPA-inoculated mice, challenged or not with DENV2, were incubated in triplicate with 2 μg of E or NS1 peptides (pooled or individually). Non-stimulated and concanavalin A (Con A, 5 μg/mL) stimulated cells were used as negative and non-specific positive controls, respectively. After an 18-h stimulation period at 37°C in 5% CO_2_, cells were discarded, and plates were incubated for 2 h at 37°C with 2 μg/mL anti-IFN-γ biotinylated detection antibody. Plates were then incubated with streptavidin-horseradish peroxidase conjugate for 1 h at room temperature (diluted 1:100). Finally, plates were washed and the spots were revealed by adding the AEC substrate set (BD Biosciences) at room temperature. The reaction was stopped by washing plates with distilled water. Spots were counted in the automated immunospot reader (AMBRIEX, Cellular Technology Ltd) at the ELISPOT Multi-User Platform (Fiocruz). Positivity was established by using a low stringent approach, in which ≥ 5 spot-forming cells (SFC) per 5 × 10^5^ cells were considered positive after subtraction of the number of spots detected in the respective non-stimulated cells, and as long as above the number observed in controls (detected in cells from pcTPA-inoculated or naïve mice).

### Intracellular Cytokine Staining (ICS) Assays

Splenocytes isolated from immunized or naïve BALB/c mice (*n* = 5), infected or not with DENV2, were tested by IFN-γ and TNF-α ICS assays, using the peptides previously identified as positive by the ELISPOT analysis. A total of 2 × 10^6^ cells/well were plated in 96-well U-bottom plates. Splenocytes were stimulated with 2 μg of E- or NS1-derived peptides or with Con A, and incubated at 37°C in 5% CO_2_ for 6 h. Brefeldin A (1 μL/mL, GolgiPlug BD Biosciences) was added to the cultures after an initial stimulation period of 1 h and 30 min. Cells were then collected and washed in staining buffer (PBS, 2% FCS, 2 mM EDTA, 55 μM β-mercaptoethanol), blocked with 10% inactivated murine serum in PBS for 30 min, fixed with 4.0% paraformaldehyde in PBS for 20 min at 4°C, washed in PBS, and then maintained at 4°C. On the next day, cells were stained with pre-titrated anti-CD3 PE (clone 145-2C11, BD Biosciences), anti-CD4 PerCP or APC (clone RM4-5, BD Biosciences) and anti-CD8 FITC or PerCP (clone 33-6.7, BD Biosciences), fixed and permeabilized using Cytofix/Cytoperm solution (BD Biosciences), according to the manufacturer's instructions. Cells were then stained for intracellular cytokine detection with anti-IFN-γ Alexa Fluor 488 (clone XMG12, BioLegend) and anti-TNF-α Alexa Fluor 647 (clone MP6-XT22, BD Biosciences). All antibodies were diluted in staining buffer. Cells were incubated for 30 min at 4°C, washed and maintained in staining buffer at 4°C until the next day. Fifteen thousand cells on the lymphocyte gate were acquired in a FACSCalibur (BD Biosciences) and data were analyzed using FlowJo software v.10 (TreeStar).

### Statistical Analysis

All statistical differences were assessed using GraphPad Prism software v6.0, applying a minimum level of significance of 95%. Statistical significance was evaluated by the non-parametric Mann-Whitney test for ELISPOT and ICS assays. Morbidity comparisons were made using one-way ANOVA with Bonferroni correction. Survival rates were evaluated using the Log-Rank statistical test.

## Results

### Protective Efficacy of pE1D2 and pcTPANS1 DNA Vaccines in BALB/c Mice

Protection conferred by the DNA vaccines based on the ectodomain of the envelope (pE1D2) and the NS1 (pcTPANS1) proteins was evaluated in BALB/c mice, after a lethal DENV2 challenge, inoculated by the i.c. route. Mice were followed for 21 days after challenge and compared to animals inoculated with the control plasmid pcTPA or to non-immunized mice, both also challenged with DENV2 (Table 1). Ninety-five percent of pE1D2-immunized mice survived the challenge, and only 15% presented clinical signs of infection. Immunization with pcTPANS1 also generated a high protection level of 85% survival, and only 20% of mice displayed morbidity. On the other hand, both pcTPA-inoculated and non-immunized mice (DENV2 group) presented low survival (30 and 20%, respectively) and high morbidity rates (90%; Table 1).

**Table 1:** Survival and morbidity of BALB/c mice immunized with the DNA vaccines pE1D2 and pcTPANS1 after lethal challenge with DENV2.

**Experimental group**	**Survival**	**Morbidity**	**Degree of morbidity**
pE1D2	19 (95%)	3 (15%)	0 ± 0.9
pcTPANS1	17 (85%)	4 (20%)	0 ± 1.5
pcTPA	6 (30%)	18 (90%)	3 ± 1.5
DENV2 (non-immunized)	4 (20%)	18 (90%)	4 ± 1.3

*Survival and morbidity, followed 21 days after virus challenge, were represented as the absolute number of individual events in the experimental group (n = 20) and the percentage it represents (in parentheses). The degree of morbidity was represented as the median ± standard deviation of the mean*.

The protection elicited by the DNA vaccines was also measured by analyzing the signs of infection in the different experimental groups through a clinical score scale from 0 to 4. Following such analysis, both pE1D2 and pcTPANS1-immunized mouse groups presented the median clinical score of 0. In contrast, animals inoculated with the control pcTPA or non-immunized mice and challenged with DENV2 presented morbidity degrees of 3 and 4, respectively (Table 1). Overall, taking together survival and morbidity rates, our results highlight the protective potential of these DNA vaccines.

### The Impact of CD4^+^ and CD8^+^ T Lymphocytes on the Survival of Immunized Mice After Lethal Virus Challenge

**Figure 1:**
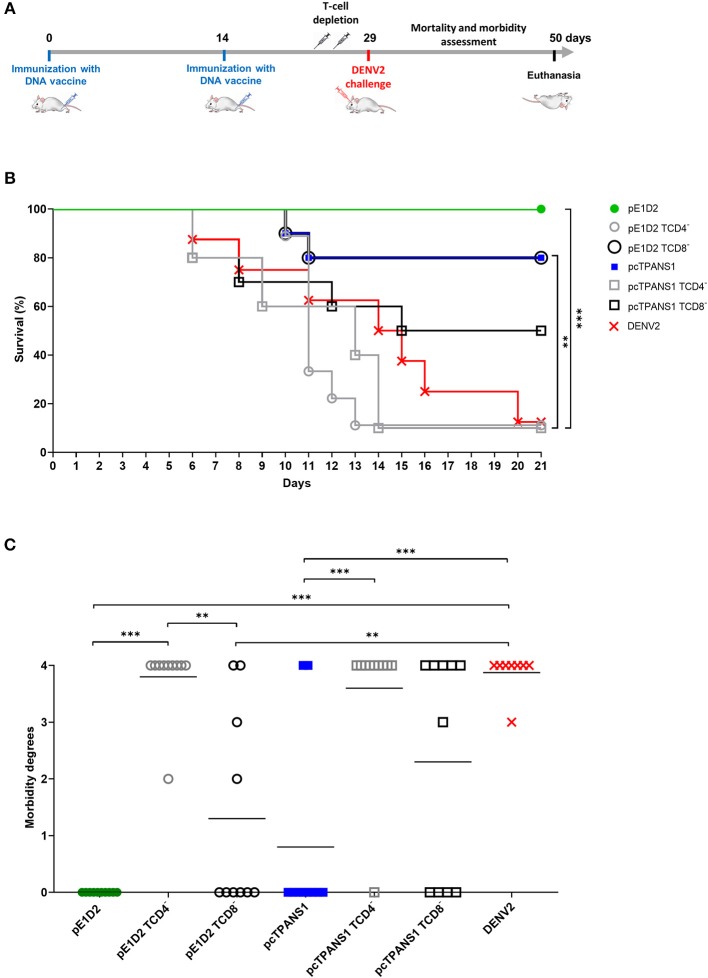
Effects of CD4^+^ and CD8^+^ T lymphocyte depletion on survival and morbidity in animals immunized with pE1D2 or pcTPANS1 and challenged with DENV2. Schematic timeline representation of the experiment **(A)**. BALB/c mice intramuscularly immunized with pE1D2 or pcTPANS1 (*n* = 10) were intraperitoneally inoculated with anti-CD4 or anti-CD8 antibodies at days 4 and 2 prior to intracerebral virus challenge (40 x LD_50_ of a neuroadapted DENV2). The control group (DENV) consisted of non-immunized mice (*n* = 8) inoculated with DENV2. All experimental groups were monitored for 21 days after challenge to record the survival rates **(B)** and degree of morbidity **(C)**. Asterisks indicate significant differences using Log-Rank (Mantel-Cox) test in **(B)** or one-way ANOVA test in **(C)** (*p < 0.05; **p < 0.001; ***p < 0.0001). Lines in **(C)** represent the median of each group.

We evaluated the contribution of T cell populations on the vaccine-induced protective immunity against DENV2 by treating pE1D2- and pcTPANS1-immunized BALB/c mice with anti-CD4 or anti-CD8 antibodies. On the challenge day, the success of the depletion protocol was confirmed (Supplementary Figure 1). Survival rates and signs of infection in immunized and treated mice were daily monitored for 21 days after DENV2 challenge. The schematic timeline with immunization, depletion and virus challenge is presented in Figure 1A.

In the pE1D2-immunized group, depletion of CD8^+^ cells resulted in a small reduction of survival rate, from 100 to 80%, with no statistical significance comparing to non-depleted animals (Figure 1B). However, CD8^+^ T cell depletion had an impact on the clinical signs of infection. In addition to mice that succumbed to infection (degree 4), other animals presented morbidity degrees ranging from 2 to 3 (Figure 1C). On the other hand, protection provided by pE1D2 was completely abolished after CD4^+^ T cells depletion. Similar to the control of non-immunized mice (DENV2 group), only 10% of pE1D2-vaccinated and anti-CD4-treated mice survived the virus challenge, which was statistically different from non-depleted or CD8^+^ T-cell depleted groups (Figure 1B). Furthermore, the only animal that survived virus infection presented morbidity degree of 2 (Figure 1C).

Similar results were observed in animals immunized with pcTPANS1 and depleted from CD4^+^ or CD8^+^ T lymphocytes. Although not statistically significant, the survival rate of animals immunized with pcTPANS1 decreased from 80 to 50% when depleted from CD8^+^ T cells, while the impact of CD4^+^ T cell depletion was more significant, abolishing protection almost completely (only 10% survival; Figure 1B). Depletion of CD8^+^ T cells also increased morbidity, as more animals showed clinical signs of DENV infection, even though this difference was not statistically significant when compared to the pcTPANS1 non-depleted group (Figure 1C).

### T Cell Epitope Map of E and NS1 Antigens by ELISPOT Assays in BALB/c Vaccinated Animals

After we observed that protection induced in BALB/c mice immunized with the pE1D2 and pcTPANS1 DNA vaccines depends on CD4^+^ and CD8^+^ T cells, we decided to identify the peptides contained in the E and NS1 proteins which were immunogenic in vaccinated animals. For this purpose, we used two synthetic peptide libraries spanning the ectodomain of the E protein and the whole sequence of NS1 protein from the dengue serotype 2, strain New Guinea C. Each peptide is 15 amino acids in length with an overlap of 11 amino acid residues. Immunogenicity of E and NS1-derived epitopes was assessed by IFN-γ ELISPOT assays, performed initially with peptide pools (8–10 peptides each) used to stimulate splenocytes from vaccinated and control animals, with cells also pooled in the different groups. After detection of positive pools, peptides were individually tested on splenocytes pooled from the different experimental groups and then evaluated with cells from individual animals. When two positive detected peptides were adjacent, we chose the peptide that generated the highest magnitude of the response. The initial ELISPOT analyses were performed using a low stringency parameter in which the response magnitude of ≥ 5 SFC per 5 × 10^5^ cells were considered positive, after subtraction of the number of spots detected in non-stimulated cells and as long as above the observed in pcTPA-inoculated or naïve control groups. Concanavalin A was used as a non-specific positive control.

**Figure 2:**
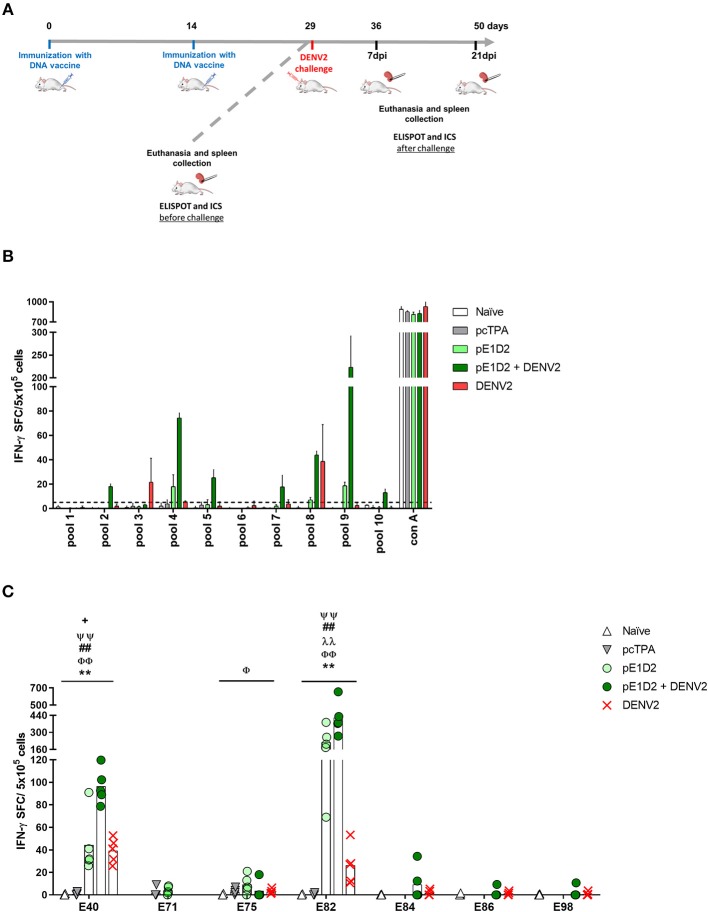
T cell E-derived peptide identification in pE1D2-immunized BALB/c mice by IFN-γ ELISPOT assays. Schematic timeline representation of the experiment **(A)**. Splenocytes isolated from pE1D2-immunized BALB/c mice (*n* = 3, pooled), challenged or not with a neuroadapted DENV2 strain (NGC), were stimulated with E-derived peptides for 18 h, and the number of cells producing IFN-γ was measured by ELISPOT assay. Peptides and cells were evaluated in pools **(B)** and positive peptides were confirmed by tests performed with cells from individual mice (*n* = 5) **(C)**. ELISPOT assays were performed using splenocytes collected 15 days following DNA inoculation (gray and light green bars/dots) or 21 **(B)** and 7 days post-infection **(C)** (dark green bars/dots). Cells from naïve or pcTPA-inoculated mice were used as negative controls **(B,C)**. The horizontal dotted lines represent the cut-off selection point (≥5 SFC/5 × 10^5^ cells) **(B)**. Bars represent the mean plus standard deviation of triplicate data in **(B)** and the means of each group in **(C)**. Symbols represent significant differences between groups, using non-parametric two-tailed Mann-Whitney statistical tests: * pcTPA- vs. pE1D2-inoculated mice; # naïve animals vs. pE1D2-immunized mice after DENV2 challenge; **+** pE1D2-immunized mice vs. pE1D2-immunized animals after DENV2 challenge; Φ naïve animals vs. DENV2 challenged mice; **λ** pE1D2-immunized mice vs. DENV2 challenged animals; Ψ pE1D2-immunized animals challenged with DENV2 vs. DENV2 challenged mice. One symbol: *p* < 0.05; two symbols: *p* < 0.001; three symbols: *p* < 0.0001.

In order to investigate whether the immunodominant pattern of the E- and NS1-derived peptides could be altered after virus infection, we also evaluated the IFN-γ response with cells collected from animals immunized with the pE1D2 or pcTPANS1 and challenged with DENV2. The schematic timeline with immunization, virus challenge and euthanasia is presented in Figure 2A.

The screening of E-derived peptide pools recognized by splenocytes from pE1D2-immunized BALB/c mice revealed 3 positive sets without the DENV2 challenge (pools 4, 8, and 9) and 4 more sets after virus infection (pools 2, 5, 7, and 10) for IFN-γ production (Figure 2B). Positive pools were then selected for individual peptide screening. Peptides E40, E71, E75, E82, and E83 were considered positive without the virus challenge (Supplementary Figure 2) by applying the same selection criteria as before and comparing to control (cells from pcTPA-inoculated animals). Given the adjacent localization of peptides E82 and E83 and the higher magnitude of response induced by the peptide E82, this peptide was selected for further analysis. The screening of individual peptides after the DENV2 challenge revealed that not all positive pools presented peptides able to stimulate IFN-γ production. Results reinforced peptides E40 and E82 as the most immunogenic, with the further addition of peptides E75, E84, E86, and E98 (Supplementary Figure 2). To further confirm the immune relevance of these peptides, we performed *in vitro* stimulation tests with cells from individual BALB/c mice. Results showed that peptides E40 and E82 significantly stimulated IFN-γ production in splenocytes collected from pE1D2-immunized mice when comparing to cell spots of pcTPA-inoculated or naïve animals stimulated with the same peptides (Figure 2C). These peptides were immunogenic in vaccinated-only mice as well as in immunized animals challenged with DENV2, although the response was higher after virus infection. Besides, both peptides were also able to elicit IFN-γ production in splenocytes collected from non-immunized mice infected with DENV2, although in a lower magnitude when compared to the pE1D2-immunized groups (Figure 2C). In contrast, the responses elicited by peptides E71, E75, E84, E86, and E98 were not significantly different from the negative controls. As expected, stimulation with Con A in all ELISPOT assays performed with cells from pE1D2-vaccinated or control animals induced high IFN-γ response.

**Figure 3:**
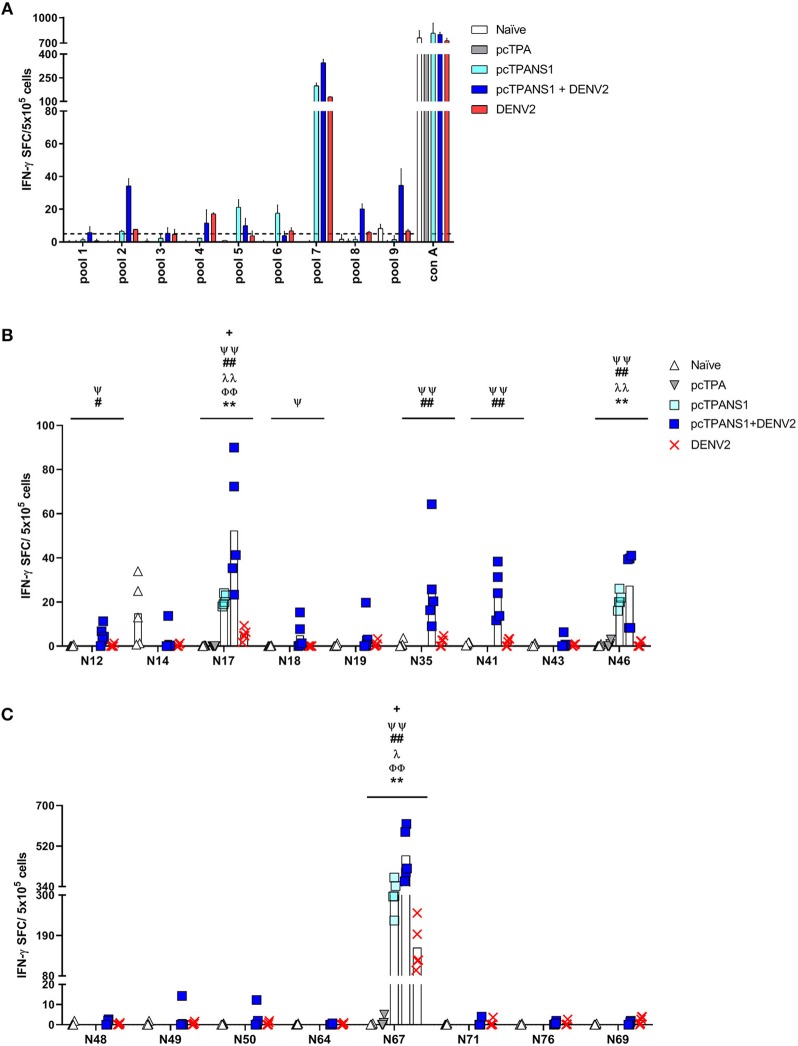
T cell NS1-derived peptide identification in pcTPANS1-immunized BALB/c mice by IFN-γ ELISPOT assays. Immunizations, virus challenge and euthanasia were outlined in Figure 2A. Splenocytes isolated from pcTPANS1-immunized BALB/c mice (*n* = 3, pooled), challenged or not with DENV2, were stimulated with NS1-derived peptides for 18 h, and the number of cells producing IFN-γ was measured by ELISPOT assay. Peptides and cells were evaluated in pools **(A)** and positive peptides were confirmed by tests performed with cells from individual mice (*n* = 5) **(B,C)**. ELISPOT assays were performed using splenocytes collected 15 days following DNA inoculation (gray and light blue bars/dots) or 21 **(A)** and 7 days post-infection **(B,C)** (dark blue bars/dots). Cells from naïve or pcTPA-inoculated mice were used as negative controls. The horizontal dotted lines represent the cut-off selection point (≥5 SFC/5 × 10^5^ cells) **(B,C)**. Bars represent the mean plus standard deviation of triplicate data in **(A)** and the means of each group in **(B,C)**. Symbols represent significant differences between groups, using non-parametric two-tailed Mann-Whitney statistical tests: * pcTPA- vs. pcTPANS1-inoculated mice; # naïve animals vs. pcTPANS1-immunized mice after DENV2 challenge; **+** pcTPANS1-immunized mice vs. pcTPANS1-immunized animals after DENV2 challenge; Φ naïve animals vs. DENV2 challenged mice; λ pcTPANS1-immunized mice vs. DENV2 challenged animals; Ψ pcTPANS1-immunized animals challenged with DENV2 vs. DENV2 challenged mice. One symbol: *p* < 0.05; two symbols: *p* < 0.001; three symbols: *p* < 0.0001.

The same screening analysis was used to evaluate the immunogenicity of NS1-derived peptides in the context of the pcTPANS1 DNA vaccine. Four peptide pools (pools 2, 5, 6, and 7) were able to elicit an IFN-γ response with magnitudes higher than or equal to 5 SFC per 5 × 10^5^ cells in splenocytes collected from pcTPANS1-immunized mice without virus challenge, and three other pools (pools 4, 8, and 9) were identified as positive after dengue infection (Figure 3A). Individual peptide screening tests revealed 4 immunogenic peptides: N17, N46, N66, and N67 (Supplementary Figure 3). Considering the position of each immunogenic region, we selected peptides N17, N46, and N67 for the assays performed with cells from individual BALB/c mice without virus challenge. The IFN-γ responses elicited by all these three NS1-derived peptides were statistically higher when comparing cells from pcTPANS1-vaccinated and pcTPA-inoculated mice (Figures 3B,C). The response against these NS1-derived peptides also increased after virus challenge, mainly regarding peptides N17 and N67. Besides, we observed that the number of immunogenic peptides increased after virus challenge. Peptides N12, N14-20, N35, N41, N43, N46-50, N64, N66, N67, N71, N72, N76, and N69 were considered positive by applying our selection criteria (Supplementary Figure 3). Nevertheless, the ELISPOT assays performed with cells from individual BALB/c mice and the selected NS1-derived peptides revealed that only 7 peptides (N12, N17, N18, N35, N41, N46, and N67) were able to stimulate an IFN-γ response significantly higher in splenocytes collected from mice immunized with pcTPANS1 and challenged with DENV2, when comparing to cells obtained from naïve or only DENV2 infected animals (Figures 3B,C). Interestingly, only the N17 and N67 peptides were able to induce production of IFN-γ in cells collected from non-immunized animals challenged with DENV2. As expected, stimulation with Con A in all ELISPOT assays performed with cells from pcTPANS1-vaccinated or control animals induced high IFN-γ response.

### Phenotyping T Cells That Recognized the Immunogenic E- and NS1-Derived Peptides

We next characterized the peptide-specific responses by investigating IFN-γ and TNF-α production in ICS assays performed with splenocytes collected from BALB/c mice immunized with the pE1D2 or pcTPANS1 DNA vaccines, before and after virus challenge. Following a brief stimulation with the peptides selected by our ELISPOT assays, we examined the intracellular cytokine production by CD4^+^ or CD8^+^ T cell populations. The flow cytometry gate strategy applied to this analysis is described in Supplementary Figures 4, 5.

**Figure 4:**
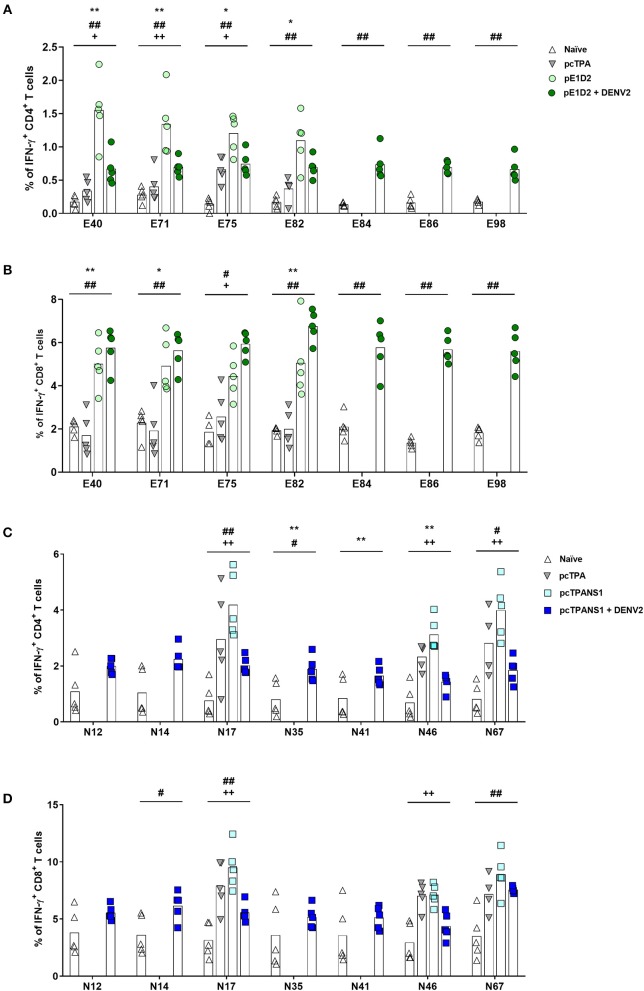
Frequencies of IFN-γ-producing T cells after stimulation with E- or NS1-derived peptides by ICS assays. Immunizations, virus challenge, and euthanasia were outlined in Figure 2A. Splenocytes isolated from BALB/c mice immunized with pE1D2 or pcTPANS1 (*n* = 5), challenged or not with DENV2, were stimulated with E- **(A,B)** or NS1-derived peptides **(C,D)** for 6 h and INF-γ production was detected by ICS assay. Peptides were selected according to previous ELISPOT results, and splenocytes were harvested 15 days following DNA inoculation or 7 days post-infection. Numbers indicate CD4^+^ **(A,C)** or CD8^+^ T cells **(B,D)** producing INF-γ as a percentage of total splenocytes. Cells from naïve or pcTPA-inoculated mice were used as negative controls. Bars represent the means of each group. Symbols represent significant differences between groups, using non-parametric two-tailed Mann-Whitney statistical tests: * pcTPA- vs. pE1D2- or pcTPANS1-inoculated mice; # naïve animals vs. pE1D2- or pcTPANS1-immunized mice after DENV2 challenge; **+** pE1D2- or pcTPANS1-immunized mice vs. pE1D2- or pcTPANS1-immunized animals after DENV2 challenge. One symbol: *p* < 0.05; two symbols: *p* < 0.001; three symbols: *p* < 0.0001.

The ICS performed with E-derived peptides revealed the involvement of both CD4^+^ and CD8^+^ T cells in IFN-γ production by splenocytes collected from pE1D2-immunized mice (Figures 4A,B). Almost all tested peptides induced IFN-γ responses in cells obtained from vaccinated animals significantly higher when compared to the response observed in lymphocytes from pcTPA-inoculated mice. The peptide E40 elicited the highest number of CD4^+^ T cells producing IFN-γ (1.5%), an increase of 4.5 times compared to the control group (Figure 4A). This peptide also led to high frequencies of IFN-γ-producing CD8^+^ T cells collected from immunized mice, although peptide E82 was the most immunogenic, with a 5% IFN-γ^+^ CD8^+^ T cell frequency (2.5-fold increase compared to cells from pcTPA-inoculated animals; Figure 4B). In general, the IFN-γ response in CD4^+^ T cells upon stimulation with all the selected peptides decreased after virus infection (average frequency from 1.5 to 0.7%, with a 2.1-fold decrease), while the frequencies of IFN-γ^+^ CD8^+^ T cells increased (average frequency of 4.7–6.0%, with a 1.3-fold increase; Figures 4A,B).

We further investigated the immune response elicited by the NS1-derived peptides by characterizing IFN-γ production by ICS assays performed with splenocytes from pcTPANS1-vaccinated mice. We also observed the production of this cytokine by CD4^+^ and CD8^+^ T cells. Although CD4^+^ T lymphocytes responded to almost all tested peptides, CD8^+^ T cells responded only to some of them (peptides N14, N17, and N67) after the dengue infection (Figures 4C,D). In terms of statistical significance, the peptide N67 was more immunogenic leading to a 4% IFN-γ^+^ CD4^+^ T cell frequency (1.4-fold increase compared to the control pcTPA; Figure 4C). Peptide N17 also induced high frequencies of CD4^+^ T cells expressing IFN-γ, although not statistically significant compared to the pcTPA group. Overall, in the context of the pcTPANS1 DNA vaccine, the percentage of T cells expressing IFN-γ seemed to have been reduced after the infection with DENV2 (Figures 4C,D).

**Figure 5:**
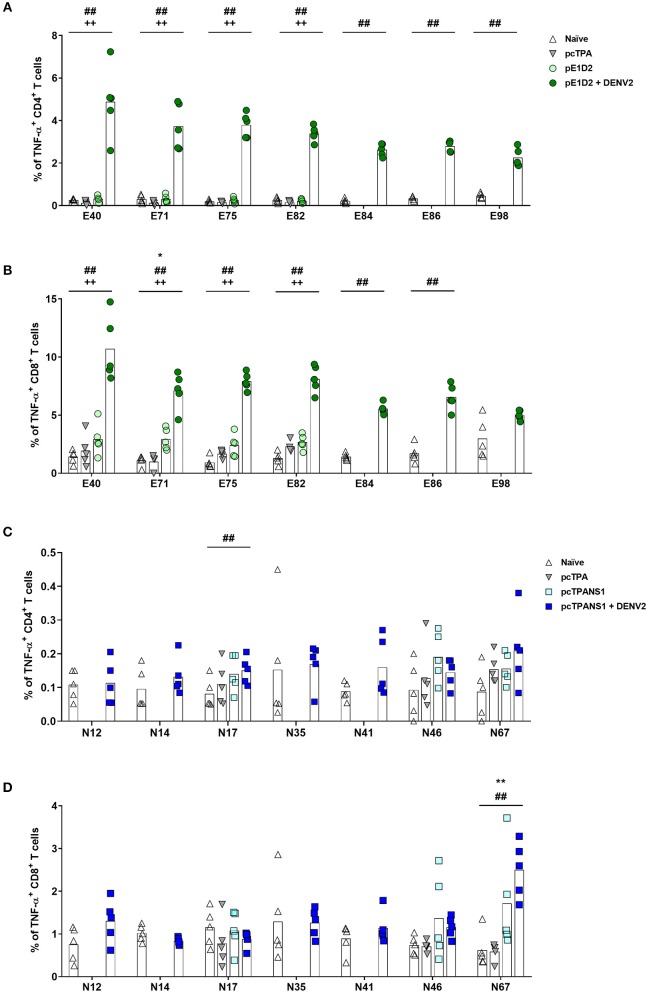
Frequencies of TNF-α-producing T cells after stimulation with E- or NS1-derived peptides by ICS assays. Immunizations, virus challenge, and euthanasia were outlined in Figure 2A. Splenocytes isolated from BALB/c mice immunized with pE1D2 or pcTPANS1 (*n* = 5), challenged or not with DENV2, were stimulated with E- **(A,B)** or NS1-derived peptides **(C,D)** for 6 h and TNF-α production was detected by ICS assay. Peptides were selected according to previous ELISPOT results, and splenocytes were harvested following DNA inoculation or 7 days post infection. Numbers indicate CD4^+^ **(A,C)** or CD8^+^ T cells **(B,D)** producing TNF-α as a percentage of total splenocytes. Cells from naïve or pcTPA-inoculated mice were used as negative controls. Bars represent the means of each group. Symbols represent significant differences between groups, using non-parametric two-tailed Mann-Whitney statistical tests: * pcTPA- vs. pE1D2- or pcTPANS1-inoculated mice; # naïve animals vs. pE1D2- or pcTPANS1-immunized mice after DENV2 challenge; + pE1D2- or pcTPANS1-immunized mice vs. pE1D2- or pcTPANS1-immunized animals after DENV2 challenge. One symbol: *p* < 0.05; two symbols: *p* < 0.001; three symbols: *p* < 0.0001.

We also evaluated TNF-α production upon stimulation with the same E- and NS1-derived peptides. After the infection with DENV2, all E-derived peptides induced a TNF-α response in CD4^+^ and CD8^+^ T cells collected from pE1D2-vaccinated animals, except for CD8^+^ T lymphocytes stimulated with the peptide E98 (Figures 5A,B). Without the virus challenge, on the other hand, an increase in the TNF-α production was not statistically detected, except for CD8^+^ T cells upon stimulation with peptide E71. Concerning the pcTPANS1 vaccine, a remarkable difference in levels of TNF-α production was not detected by the ICS assays using cells from vaccinated animals, challenged or not with DENV2 (Figures 5C,D). The only peptides able to induce TNF-α expression were N17 in CD4^+^ T cells and N67 in CD8^+^ T cells.

Results from the screening of E- and NS1-derived peptides and production of IFN-γ and TNF-α are summarized in Table 2, as well as their amino acid sequences and location on the E and NS1 proteins.

**Table 2:** Characteristics and summary results of E and NS1 positive peptides.

			**IFN-γ ELISPOT Score**			**% of IFN-γ^**+**^ T cells**				**% of TNF-α^**+**^ T cells**			
**Envelope peptides**	**Sequence**	**Location**	**pE1D2**	**pE1D2 ^+^DENV2**	**DENV2**	**pE1D2**		**pE1D2** **^+^DENV2**		**pE1D2**		**pE1D2** **^+^** **DENV2**	
						**CD4^**+**^**	**CD8^**+**^**	**CD4^**+**^**	**CD8^**+**^**	**CD4^**+**^**	**CD8^**+**^**	**CD4^**+**^**	**CD8^**+**^**
E40	KHGKEIKITPQSSIT	157–171	^++^	^+++^	^++^	1.55	5.01	0.67	5.76	0.32	2.94	4.89	10.7
E71	GHLKCRLRMDKLQLK	281–295	NS	—	—	1.34	4.92	0.69	5.63	0.31	2.92	3.73	7.11
E75	MSYSMCTGKFKVVKE	297–311	NS	NS	NS	1.21	4.45	0.75	5.94	0.25	2.4	3.79	7.91
E82	QYEGDGSPCKIPFEI	325–339	^++++^	^++++^	^++^	1.1	5.06	0.69	6.76	0.22	2.67	3.39	8.12
E84	CKIPFEIMDLEKRHV	333–347	—	NS	NS	—	—	0.74	5.77	—	—	2.62	5.55
E86	DLEKRHVLGRLITVN	341–355	—	NS	NS	—	—	0.69	5.68	—	—	2.8	6.55
E98	QLKLNWFKKGSSIVI	386–400	—	NS	NS	—	—	0.66	5.6	—	—	2.25	5.01
**NS1 peptides**	**Sequence**	**Location**	**pcTPANS1**	**pcTPANS1** **^+^** **DENV2**	**DENV2**	**pcTPANS1**		**pcTPANS1** **^+^** **DENV2**		**pcTPANS1**		**pcTPANS1** **^+^** **DENV2**	
						**CD4**^**+**^	**CD8**^**+**^	**CD4**^**+**^	**CD8**^**+**^	**CD4**^**+**^	**CD8**^**+**^	**CD4**^**+**^	**CD8**^**+**^
N12	AIQKAHEEGICGIRS	45–59	—	^+^	NS	—	—	2.0	5.55	—	—	0.11	1.3
N14	GICGIRSVTRLENLM	53–67	—	NS	NS	—	—	2.24	6.15	—	—	0.13	0.84
N17^*^	NLMWKQITPELNHIL	65–79	^++^	^+++^	^+^	4.19	9.5	2.02	5.56	0.14	1.08	0.15	0.88
N35^*^	GPETAECPNTNRAWN	137–151	—	^++^	NS	—	—	1.89	5.17	—	—	0.17	1.26
N41	GVFTTNIWLKLREKQ	161–175	—	^++^	NS	—	—	1.65	5.13	—	—	0.16	1.14
N46	SKLMSAAIKDNRAVH	181–195	^++^	^++^	NS	3.13	7.12	1.43	4.38	0.19	1.37	0.14	1.16
N67	AGPWHLGKLEMDFDF	265–279	^++++^	^++++^	^++++^	3.99	8.92	1.85	7.56	0.15	1.71	0.21	2.5

The location of positive peptides was determined according to the amino acid sequence of E and NS1 proteins from NGC DENV2. Results from IFN-γ ELISPOT assays performed with cells from individual mice are displayed according to the following arbitrary scale: (+): ≥5 to 20; (++): 21 to 50, (+++): 51 to 100, (++++): ≥100 SFC/5 × 10^5^ cells. % of IFN-γ^+^ and TNF-α^+^ T cells are represented as mean ICS frequencies. (^*^) Peptides also positive in IFN-γ ELISPOT with cells from C57BL/6 mice. (—): means non-existent data; (NS): non-statistical.

### T Cell Epitope Map of E and NS1 Proteins in C57BL/6 Mice

In addition to studies with BALB/c animals, we also investigated the immunodominance of E- and NS1-derived peptides in the context of a different MHC haplotype expression. For this purpose, we immunized C57BL/6 mice with pE1D2 and pcTPANS1 DNA vaccines and evaluated the IFN-γ response by ELISPOT assays using the same E and NS1 peptide libraries. Following the low stringent analysis performed before, peptide pools that elicited a magnitude of response higher or equal to 5 SFC per 5 × 10^5^ cells were considered positive.

**Figure 6:**
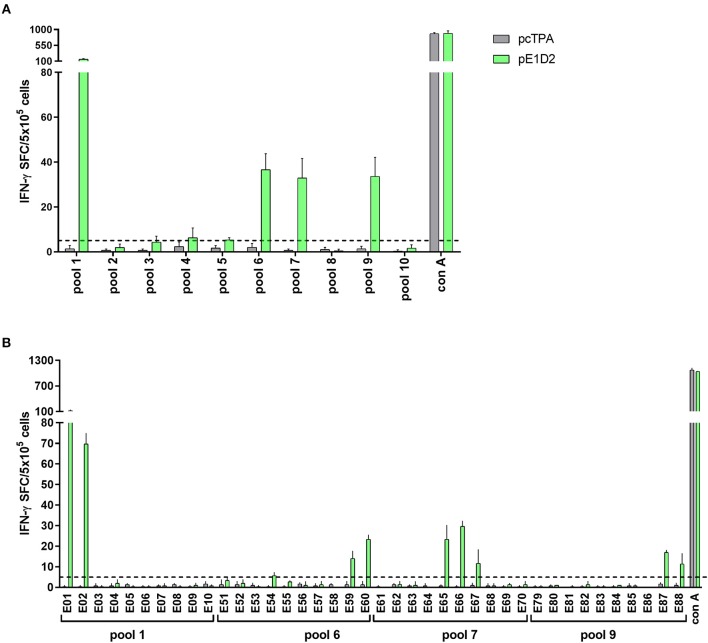
T cell E-derived peptide identification in pE1D2-immunized C57BL/6 mice by IFN-γ ELISPOT assays. Splenocytes from pE1D2-immunized C57BL/6 mice (*n* = 3, pooled) were harvested 15 days following DNA inoculation, stimulated with E-derived peptides for 18 h, and the number of cells producing IFN-γ was measured by ELISPOT assay. Peptides and cells were evaluated in pools **(A)** and positive pools were selected for individual peptide screening **(B)**. Cells from pcTPA-inoculated mice were used as a negative control. The horizontal dotted lines represent the cut-off selection point (≥ 5 SFC/5 × 10^5^ cells). Bars represent the mean plus standard deviation of triplicate data.

A total of 4 E-derived peptide pools were identified as positive for stimulating IFN-γ production (pools 1, 6, 7, and 9) and were selected for individual peptide screenings (Figure 6A). Peptides E01, E02, E59, E60, E65-67, E87, and E88 were able to induce an IFN-γ response in splenocytes collected from animals vaccinated with pE1D2 (Figure 6B).

**Figure 7:**
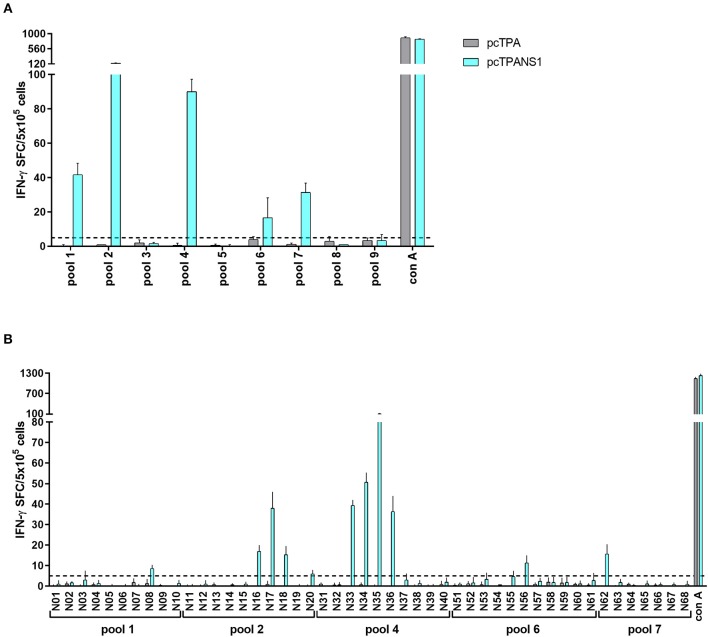
T cell NS1-derived peptide identification in pcTPANS1-immunized C57BL/6 mice by IFN-γ ELISPOT assays. Splenocytes from pcTPANS1-immunized C57BL/6 mice (*n* = 3, pooled) were harvested 15 days following DNA inoculation, stimulated with NS1-derived peptides for 18 h, and the number of cells producing IFN-γ was measured by ELISPOT assay. Peptides and cells were evaluated in pools **(A)** and positive pools were selected for individual peptide screening **(B)**. Cells from pcTPA-inoculated mice were used as a negative control. The horizontal dotted lines represent the cut-off selection point (≥5 SFC/5 × 10^5^ cells). Bars represent the mean plus standard deviation of triplicate data.

Similarly, 5 NS1-derived peptide pools (pools 1, 2, 4, 6, and 7) were able to stimulate IFN-γ production in cells collected from C57BL/6 mice immunized with the pcTPANS1 DNA vaccine (Figure 7A). Individual peptide screenings revealed that peptides N08, N16-18, N20, N33-36, N56, and N62 elicited IFN-γ expression in splenocytes obtained from pcTPANS1-immunized C57BL/6 mice (Figure 7B). These results revealed that peptides N17 and N35 were immunogenic in the context of the two different evaluated MHC haplotypes, i.e., in BALB/c and in C57BL/6 mice.

## Discussion

In this study, we evaluated the contribution of CD4^+^ and CD8^+^ T cells for the protection induced in mice by DNA vaccines encoding the ectodomain of the envelope (pE1D2) and the non-structural 1 (pcTPANS1) proteins from DENV2. We also identified the T cell immunogenic peptides in vaccinated animals by the production of IFN-γ and TNF-α. We have described the DNA vaccines construction elsewhere (33, 40) and in the present work, we confirmed protection elicited in immunocompetent BALB/c mice challenged with a neuroadapted NGC DENV2. Almost all immunized animals survived virus infection with none or low morbidity degrees. The murine model of BALB/c mice inoculated with DENV2 by the i.c. route is well-established in our laboratory and provides a straightforward readout parameter for vaccine testing. Therefore, in order to expand the knowledge about the different arms of the immune system that may act on the protection against DENV, we investigated in BALB/c mice the role of the T cell response elicited by the DNA vaccines we have constructed. We observed that the CD4^+^ T lymphocytes response was essential for protection generated by both vaccines, although depletion of CD8^+^ T cells also impacted in survival and morbidity after virus challenge. In keeping with these results, we have previously shown that pcTPANS1-vaccinated animals exhibited a significant increase of activated CD4^+^ and CD8^+^ T cells in spleen and blood circulation after the DENV2 challenge (44).

Our group has also reported the importance of CD4^+^ T cells in pcTPANS1-immunized mice in a study showing that these lymphocytes in association with anti-NS1 antibodies are fundamental for protection (39). In addition, the CD4^+^ T cells induced by the pcTPANS1 vaccine seem to act by a different mechanism than helping B-cell antibody production. In fact, our previous study with adoptive transfer of CD4+ T cells combined with NS1 antiserum, both obtained from vaccinated mice, led to the protection of recipient BALB/c animals after challenge with DENV2, with survival rates not significantly different from those observed in pcTPANS1-immunized mice (39). Since recipient animals did not receive B cells, only CD4^+^ T cells and antibodies, the mechanism involved in the protection mediated by these T cells must be other than helping B lymphocytes.

On the other hand, several studies have pointed out the importance of CD8^+^ T cells in controlling DENV infection, either in mouse models or in humans (16, 17, 19, 45–47). Yet, other reports suggest the involvement of CD4^+^ T lymphocytes in the protection provided by experimental vaccines (25, 28). Yauch and colleagues showed that CD8^+^ T cells played an essential role in viral clearance, while CD4^+^ T lymphocytes were not required to control primary DENV2 infection in IFN-α/βR^−/−^ C57BL/6 mice. However, immunization with CD4^+^ T cell epitopes conferred protection by reducing viral load after challenge, thus supporting the importance of CD4^+^ T cell induction by vaccination (25). Besides, activation of cytotoxic CD4^+^ T cells has been reported after DENV infection, which seems to be associated with protective immunity (18, 48). Additionally, CD4^+^ and CD8^+^ T cell epitope reactivities against DENV2 was investigated in patients experiencing secondary DENV infection (27). Authors observed that CD8^+^ T cells preferentially targeted epitopes contained in the NS3 and NS5 proteins, while CD4^+^ T lymphocytes recognized mainly epitopes derived from the virus envelope, capsid, and NS1 proteins. Nevertheless, another study suggested that the hierarchy of immunodominance between the different DENV proteins depends on the virus serotype (47).

Mouse models have been essential in determining the T cell epitopes involved in the protection and/or the pathogenesis of dengue infections (46). Our study conducted in an immunocompetent mouse model revealed that the pE1D2 and pcTPANS1 DNA vaccines were able to induce T cell responses targeting both E and NS1 proteins. The T cell epitopes were screened by ELISPOT and ICS assays for IFN-γ production in splenocytes from vaccinated mice, using 15-mer synthetic peptide libraries with 11 amino acid residues overlapping, spanning the E and NS1 proteins. The sets of peptides of 15 amino acids length were chosen since they can efficiently stimulate both CD4^+^ and CD8^+^ T cells (49). The assays to measure IFN-γ response were selected since several reports indicate that the production of this cytokine contributes to protection against DENV in animal models as well as in humans (25, 50, 51). The screening of T cell epitopes was also assessed in C56BL/6 mice immunized with the pE1D2 and pcTPANS1 vaccines in order to investigate the T cell response in the context of a different MHC haplotype expression. However, this mouse strain is not susceptible to our neuroadapted DENV2 sample. Therefore, experiments were not performed after virus challenge.

We identified 4 and 7 E-derived epitopes in pE1D2-vaccinated BALB/c animals before and after virus challenge, respectively (Table 2). Five of these peptides (E75, E82, E84, E86, and E98) are located in domain III of the E protein. This domain is also an important target for induction of neutralizing antibodies during virus infection (23), which is another fundamental arm of the immune response against DENV and therefore an essential region for vaccine designs. Besides, peptides E40 and E82 were immunodominant in our study, leading to a significantly higher IFN-γ production in vaccinated animals, challenged or not with DENV2, as well as in mice only infected with the virus. Peptide E82 contains the sequence SPCKIPFEI, which was the first immunodominant epitope described for H-2^d^-restricted CD8^+^ T cells in DENV2 infected mice (BALB/c) (52). As far as we know, our study is the first to identify the region present in the E40 peptide as immunodominant for T cell response.

Studies using different murine models have also identified an immunogenic T-cell epitope contained in peptides E86/E87 (amino acids 345–359) (28, 53). Chen and colleagues described a CD4^+^ T cell epitope between amino acids 349–363 in BALB/c mice immunized with a tetravalent DNA vaccine based on the domain III of the DENV envelope protein (28). On the other hand, Li and colleagues observed that one epitope present in the peptide E87 (between amino acids 345–359) can stimulate IFN-γ production in cells obtained from C57BL/6j mice infected with DENV2 (53). In our study, analyzing peptides recognized by T cells from C57BL/6 mice immunized with the pE1D2 DNA vaccine, the peptide E87 was also immunogenic for induction of IFN-γ production, although the response to this peptide was not immunodominant. Thus, the region comprising E86/E87 peptides is able to stimulate lymphocytes from mice with the distinct haplotypes H-2^d^ and H-2^b^.

Further reports, using transgenic mice with human leucocyte antigen (HLA) and infected with DENV, also identified a CD8^+^ T cell epitope contained in E86/E87 sequence, in addition to another epitope present in the peptide E75, likewise immunogenic in pE1D2-immunized BALB/c animals (26, 54). Regarding human T cell recognized epitopes, most of the E epitopes mapped in pE1D2-vaccinated animals have also been identified in DENV-infected patients (E71, E75, E82, E84, E86, and E98) and in volunteers who received a live attenuated tetravalent vaccine (E75) (17, 55). Therefore, our results together with published data reveal the potential of these E-derived regions to induce a T cell response that may be involved in protection against DENV.

On the other hand, studies on NS1 are less abundant. Our mapping of NS1 epitopes identified 3 and 7 immunogenic peptides in pcTPANS1-vaccinated mice, before and after DENV2 infection, respectively (Table 2). The N67 peptide was the immunodominant epitope, inducing high IFN-γ production, in agreement with another report that evaluated the response induced in BALB/c mice immunized with an adenovirus-based vaccine containing the DENV2 NS1 gene (56). Authors identified the CD8^+^ T cell immunodominant epitope AGPWHLGKL, which is contained in the N67 peptide and is highly conserved among strains of the four DENV serotypes. In addition, a screening of T cell epitopes derived from structural and non-structural proteins of DENV3 in HLA-transgenic mice and validated using T cells from human DENV3 immune volunteers detected an epitope contained in N17/N18 peptides that activated T-cell memory (57). In our studies, the N17 peptide was immunogenic in BALB/c animals before and after virus infection, as well as in C57BL/6 mice. Another evaluation using DENV2-infected HLA-transgenic mice revealed one peptide able to induce high levels of IFN-γ, which sequence is contained in peptide N41 (54). Moreover, human studies in Nicaragua with subjects previously infected with DENV, and in volunteers who received a live attenuated tetravalent vaccine, mapped some NS1-derived T cell epitopes, including sequences contained in the peptides N17, N41, and N67 (17, 55). Besides, one report with adult patients experiencing secondary DENV infection identified CD8^+^ T cell epitopes by ELISPOT assay and ICS contained in N41 and N48 peptide sequences (27).

Apart from evaluating the IFN-γ response induced by the screened E- and NS1-derived peptides, we also investigated TNF-α production in splenocytes from immunized animals stimulated with the previously selected peptides. Only one peptide (E71) was able to induce TNF-α production in pE1D2-vaccinated BALB/c mice without virus challenge. In contrast, almost all tested E-derived peptides led to a TNF-α response after the DENV infection either in CD4^+^ or CD8^+^ T cells. Elevated levels of TNF-α and soluble TNF-α receptors have been reported in severe cases of dengue (58, 59). Since this cytokine is involved in cellular apoptosis and increased vascular permeability, its association with dengue severity has been extensively studied, reviewed by Pang et al. (60), Srikiatkhachorn et al. (61), and Kuczera et al. (62). However, the correlation between TNF-α production and the outcome of dengue disease is still uncertain. Some reports analyzing dengue-infected patients have not observed a significant difference between TNF-α levels from dengue fever and severe dengue (63–65).

Regarding the NS1 protein, we only observed a significant increase in TNF-α^+^CD8^+^ T cells from pcTPANS1-immunized mice after stimulation with the peptide N67. Interestingly, CD4^+^ and CD8^+^ T lymphocytes from pcTPANS1-vaccinated animals after virus challenge maintained ordinary TNF-α production by stimulation with almost all NS1-derived peptides. Considering the increase of TNF-α as a deleterious effect of DENV infection, our results regarding the pcTPANS1 vaccine suggest a protective effect by inhibiting the production of such cytokine. On the other hand, analysis of polyfunctional T cell responses has drawn attention for vaccine development against flavivirus, suggesting that T lymphocytes producing multiple cytokines including IFN-γ, TNF-α, CD107a, and IL-2 are the most effective in controlling the virus (17, 18, 48, 66, 67).

In the present work, we only investigated protection against homo-typic infection. It is well-known that the severe form of the dengue disease is usually associated with hetero-typic infection, and it is a consensus that a dengue vaccine must be protective against the four DENV serotypes. Therefore, other studies will be necessary in order to evaluate the cross-reaction of the identified peptides against other DENV serotypes and its association with protection or pathogenesis. Besides, it will be likewise important to investigate whether the T cell responses induced by our DNA vaccines based on the envelope and NS1 proteins are multifunctional, as well as to examine the involvement of the identified peptides in the protection conferred by these vaccines. Our previous studies with the pE1D2 and pcTPANS1 vaccines also pointed out the importance of antibodies in protection, including the production of neutralizing antibodies against the E protein. Taking together with the results shown in the present work, we believe that the efficiency of these DNA vaccines is the combination of both arms of the immune system, the humoral and cellular immune response. Consequently, the knowledge of dominant epitopes targeted by CD4^+^ and CD8^+^ T cells might be essential for elucidating the mechanism involved in protection, resulting in more effective vaccines against dengue as well as against other flaviviruses.

## Ethics Statement

This study was carried out under ethical principles in animal experimentation stated by the Brazilian College of Animal Experimentation and approved by the Ethical Committee of Animal Use of Oswaldo Cruz Institute in Fiocruz (CEUA-IOC approval ID: L039/2015).

## Author Contributions

PP, MA, SC, and AA conceived and designed the experiments. PP, MA, AP, LL, KQ, BP, and SC performed the experiments. PP, MA, AV, SC, and AA analyzed the data. PP, MA, SC, and AA wrote the paper. AV revised the paper. All authors approved the final copy of the manuscript.

### Conflict of Interest Statement

The authors declare that the research was conducted in the absence of any commercial or financial relationships that could be construed as a potential conflict of interest.

## Supplementary Material

Supplementary Figure 1 CD4^+^ and CD8^+^ T cell depletion in BALB/c mice. Representative flow cytometry dot plots displaying CD4^+^ and CD8^+^ T cells frequencies in blood samples from T-cell depleted mice. Animals treated intraperitoneally with anti-CD4 or anti-CD8 antibodies were evaluated on the challenge day to verify the success of the depletion protocol. Blood samples were stained with anti-CD3 PE, anti-CD4 APC, and anti-CD8 FITC. The CD3^+^ population was backgated on FSS x SSC dot plot to orient the lymphocyte gate. On lymphocyte gate, CD4 x CD8 dot plots were evaluated. Cells from non-depleted naïve mice were stained or incubated with isotype antibodies and were used as positive and negative control samples.

Supplementary Figure 2 Peptide screening by evaluation of IFN-γ production in splenocytes isolated from pE1D2-immunized BALB/c mice, challenged or not with DENV2. Positive E-derived peptide pools, previously evaluated by ELISPOT assay, were selected for individual peptide screening. Screening of peptides contained in pools 2, 4 and 5 **(A)**, and in pools 7, 8, 9 and 10 **(B)**. Splenocytes were isolated from BALB/c mice 15 days following the DNA inoculation (gray and light green bars) or 21 days post-infection (dark green bars), stimulated with E-derived peptides for 18 h, and the number of cells producing IFN-γ was measured by ELISPOT assay. Cells from naïve or pcTPA-inoculated mice were used as negative control. The horizontal dotted lines represent the cut-off selection point (≥5 SFC/5 × 10^5^ cells). Bars represent the mean plus standard deviation of triplicate data.

Supplementary Figure 3 Peptide screening by evaluation of IFN-γ production in splenocytes isolated from pcTPANS1-immunized BALB/c mice, challenged or not with DENV2. Positive NS1-derived peptide pools, previously evaluated by ELISPOT assay, were selected for individual peptide screening. Screening of peptides contained in pools 2, 4, 5 and 6 **(A)**, and in pools 7, 8 and 9 **(B)**. Splenocytes were isolated from BALB/c mice 15 days after the DNA inoculation (gray and light blue bars) or 21 days post-infection (dark blue bars), stimulated with NS1-derived peptides for 18 h, and the number of cells producing IFN-γ was measured by ELISPOT assay. Cells from naïve or pcTPA-inoculated mice were used as negative control. The horizontal dotted lines represent the cut-off selection point (≥5 SFC/5 × 10^5^ cells). Bars represent the mean plus standard deviation of triplicate data.

Supplementary Figure 4 IFN-γ ICS flow cytometry analysis to evaluate CD4^+^ and CD8^+^ T cell populations from pE1D2 and pcTPANS1-immunized mice, challenged or not with DENV2. Splenocytes previously stimulated with E or NS1-derived peptides were stained with anti-CD3 PE, anti-CD4 APC, and anti-CD8 PerCP followed by intracellular staining with anti-IFN-γ Alexa Fluor 488. We backgated the CD3^+^ population on an FSS x SSC dot plot to construct the lymphocyte gate. The IFN-γ-producing CD4^+^ or CD8^+^ T cells were analyzed on CD4^+^CD3^+^ gate. Staining example of CD4^+^ or CD8^+^ T cells for IFN-γ from a pE1D2-immunized mouse.

Supplementary Figure 5 TNF-α ICS flow cytometry analysis to evaluate CD4^+^ and CD8^+^ T cell populations from pE1D2 and pcTPANS1-immunized mice, challenged or not with DENV2. Splenocytes previously stimulated with E or NS1-derived peptides were stained with anti-CD3 PE, anti-CD4 PerCP, and anti-CD8 FITC followed by intracellular staining with anti-TNF-α Alexa Fluor 647. We backgated the CD3^+^ population on an FSS x SSC dot plot to construct the lymphocyte gate. The TNF-α-producing CD4^+^ or CD8^+^ T cells were analyzed on CD4^+^CD3^+^ gate. Staining example of CD4^+^ or CD8^+^ T cells for TNF-α from a pE1D2-immunized and challenged mouse.
