# Supplementary figures and images for: T Cell Responses Induced by DNA Vaccines Based on the DENV2 E and NS1 Proteins in Mice: Importance in Protection and Immunodominant Epitope Identification
Source: Front Immunol. 2019 Jul 3;10:1522. doi: 10.3389/fimmu.2019.01522 (PMC6617960; doi:10.3389/fimmu.2019.01522)

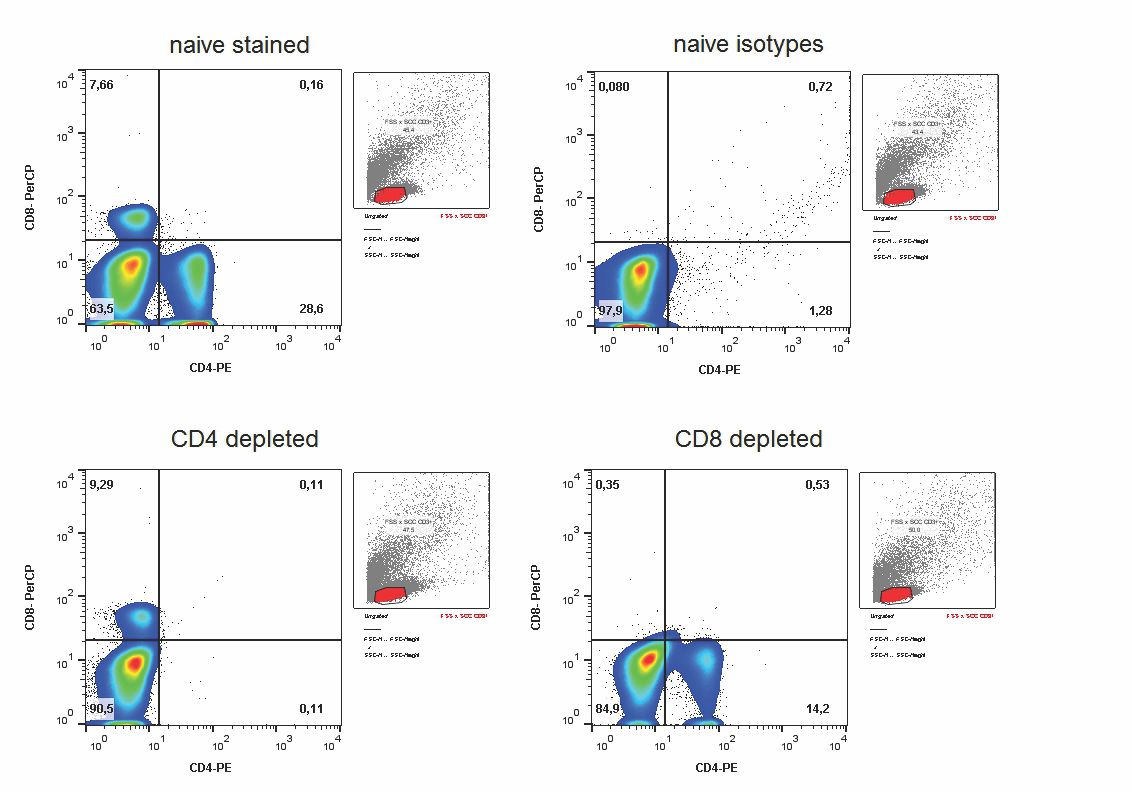

Supplement: Supplementary Figure 1 — CD4+ and CD8+ T cell depletion in BALB/c mice. Representative flow cytometry dot plots displaying CD4+ and CD8+ T cells frequencies in blood samples from T-cell depleted mice. Animals treated intraperitoneally with anti-CD4 or anti-CD8 antibodies were evaluated on the challenge day to verify the success of the depletion protocol. Blood samples were stained with anti-CD3 PE, anti-CD4 APC, and anti-CD8 FITC. The CD3+ population was backgated on FSS x SSC dot plot to orient the lymphocyte gate. On lymphocyte gate, CD4 x CD8 dot plots were evaluated. Cells from non-depleted naïve mice were stained or incubated with isotype antibodies and were used as positive and negative control samples. [file Image_1.JPEG]

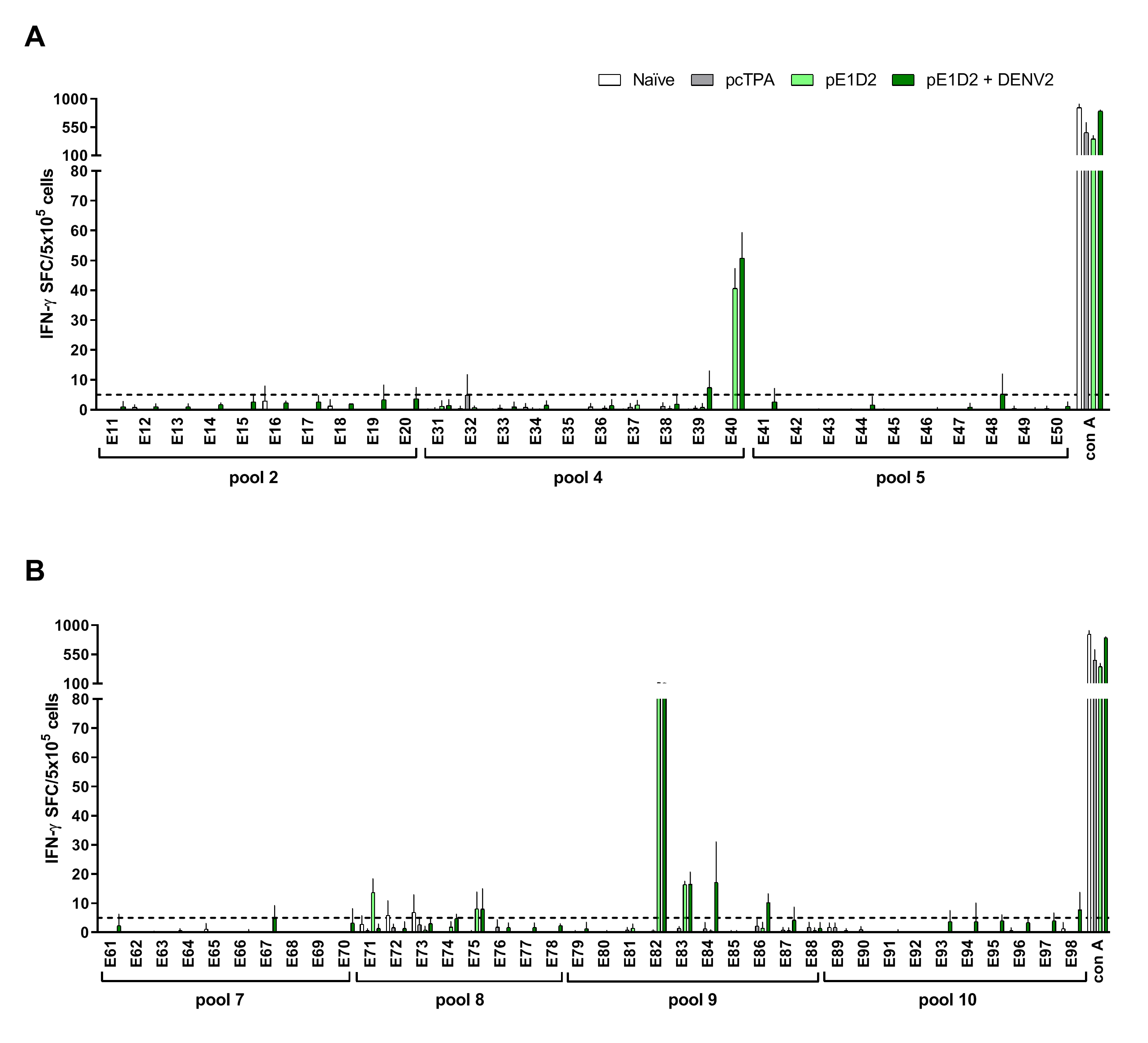

Supplement: Supplementary Figure 2 — Peptide screening by evaluation of IFN-γ production in splenocytes isolated from pE1D2-immunized BALB/c mice, challenged or not with DENV2. Positive E-derived peptide pools, previously evaluated by ELISPOT assay, were selected for individual peptide screening. Screening of peptides contained in pools 2, 4 and 5 (A), and in pools 7, 8, 9 and 10 (B). Splenocytes were isolated from BALB/c mice 15 days following the DNA inoculation (gray and light green bars) or 21 days post-infection (dark green bars), stimulated with E-derived peptides for 18 h, and the number of cells producing IFN-γ was measured by ELISPOT assay. Cells from naïve or pcTPA-inoculated mice were used as negative control. The horizontal dotted lines represent the cut-off selection point (≥5 SFC/5 × 105 cells). Bars represent the mean plus standard deviation of triplicate data. [file Image_2.tif]

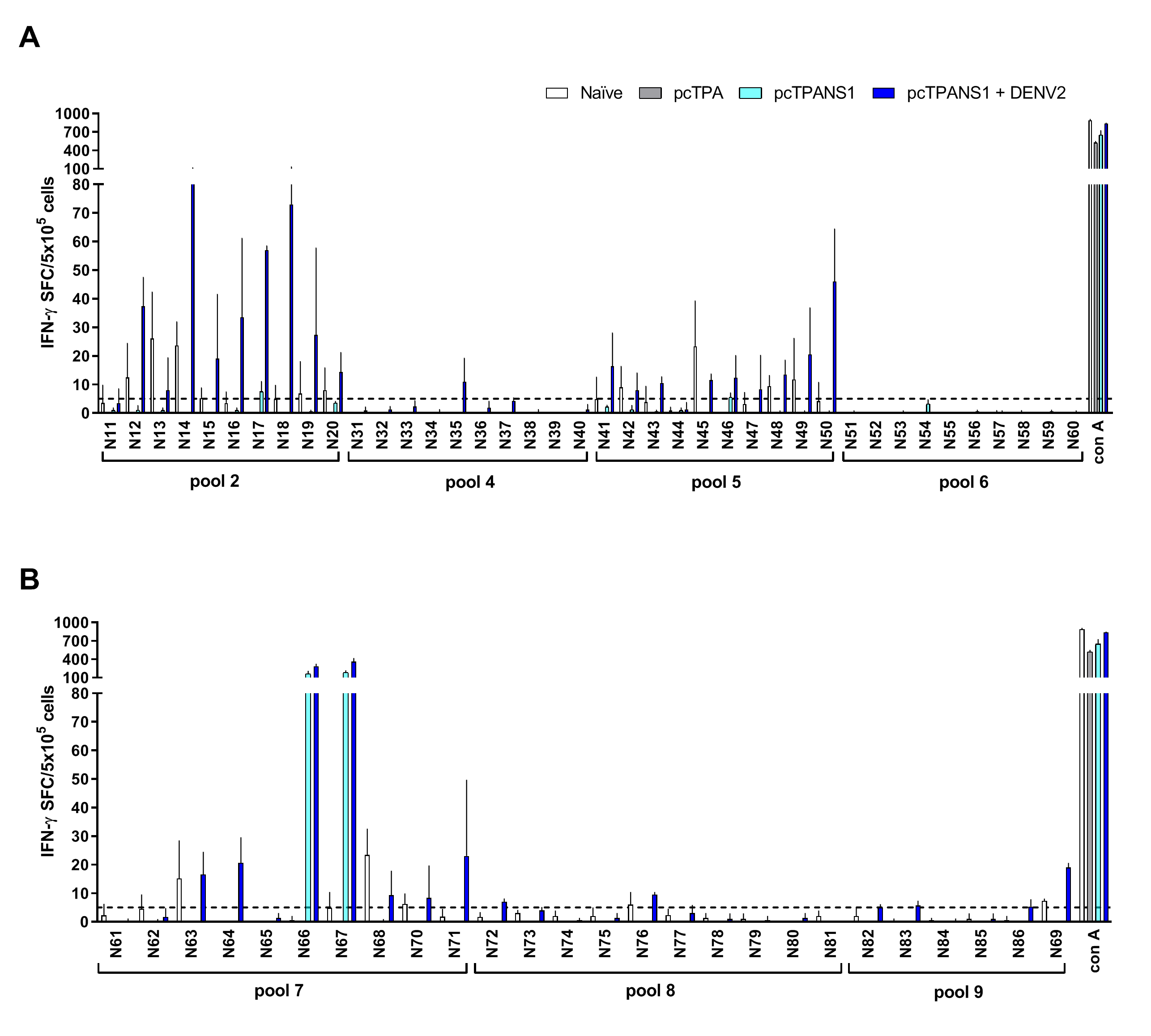

Supplement: Supplementary Figure 3 — Peptide screening by evaluation of IFN-γ production in splenocytes isolated from pcTPANS1-immunized BALB/c mice, challenged or not with DENV2. Positive NS1-derived peptide pools, previously evaluated by ELISPOT assay, were selected for individual peptide screening. Screening of peptides contained in pools 2, 4, 5 and 6 (A), and in pools 7, 8 and 9 (B). Splenocytes were isolated from BALB/c mice 15 days after the DNA inoculation (gray and light blue bars) or 21 days post-infection (dark blue bars), stimulated with NS1-derived peptides for 18 h, and the number of cells producing IFN-γ was measured by ELISPOT assay. Cells from naïve or pcTPA-inoculated mice were used as negative control. The horizontal dotted lines represent the cut-off selection point (≥5 SFC/5 × 105 cells). Bars represent the mean plus standard deviation of triplicate data. [file Image_3.tif]

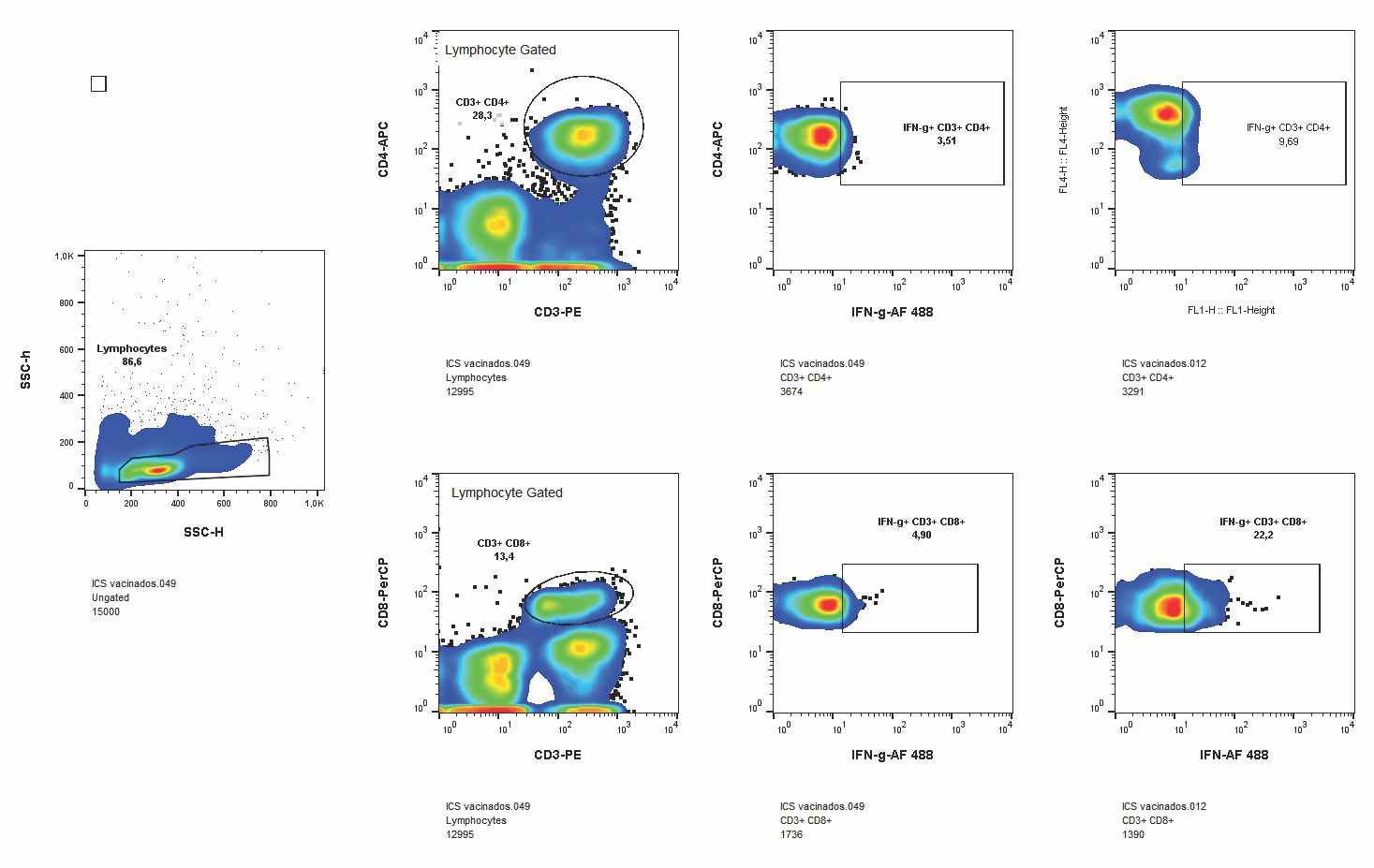

Supplement: Supplementary Figure 4 — IFN-γ ICS flow cytometry analysis to evaluate CD4+ and CD8+ T cell populations from pE1D2 and pcTPANS1-immunized mice, challenged or not with DENV2. Splenocytes previously stimulated with E or NS1-derived peptides were stained with anti-CD3 PE, anti-CD4 APC, and anti-CD8 PerCP followed by intracellular staining with anti-IFN-γ Alexa Fluor 488. We backgated the CD3+ population on an FSS x SSC dot plot to construct the lymphocyte gate. The IFN-γ-producing CD4+ or CD8+ T cells were analyzed on CD4+CD3+ gate. Staining example of CD4+ or CD8+ T cells for IFN-γ from a pE1D2-immunized mouse. [file Image_4.JPEG]

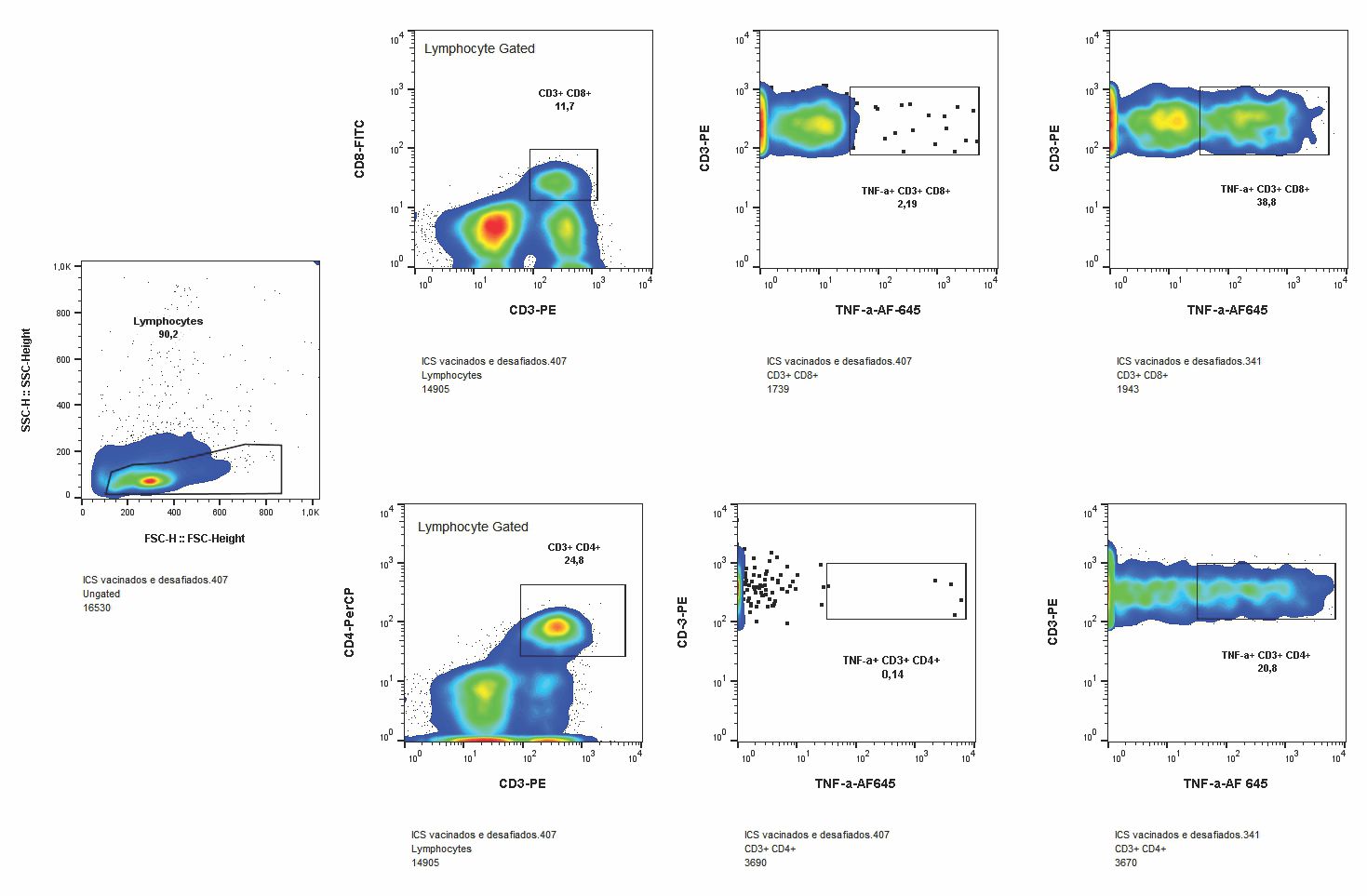

Supplement: Supplementary Figure 5 — TNF-α ICS flow cytometry analysis to evaluate CD4+ and CD8+ T cell populations from pE1D2 and pcTPANS1-immunized mice, challenged or not with DENV2. Splenocytes previously stimulated with E or NS1-derived peptides were stained with anti-CD3 PE, anti-CD4 PerCP, and anti-CD8 FITC followed by intracellular staining with anti-TNF-α Alexa Fluor 647. We backgated the CD3+ population on an FSS x SSC dot plot to construct the lymphocyte gate. The TNF-α-producing CD4+ or CD8+ T cells were analyzed on CD4+CD3+ gate. Staining example of CD4+ or CD8+ T cells for TNF-α from a pE1D2-immunized and challenged mouse. [file Image_5.JPEG]
